# Leptomeningeal Carcinomatosis: A Rare Presentation of Perforated Gastric Cancer

**DOI:** 10.7759/cureus.48775

**Published:** 2023-11-14

**Authors:** Klara Schwarzova, Xiaolong Li, Faith Adekunle, Alok Gupta

**Affiliations:** 1 Surgery, Ascension Saint Agnes Hospital, Baltimore, USA; 2 School of Medicine, American University of the Carribbean, Cupecoy, SXM; 3 General Surgery, Ascension Saint Agnes Hospital, Baltimore, USA

**Keywords:** metastatic gastric adenocarcinoma, laparoscopic treatment, leptomeningeal carcinomatosis (lmc), gastric malignancy, perforated cancer

## Abstract

Leptomeningeal carcinomatosis (LMC) or leptomeningeal metastasis is defined as metastasis to the pia mater, arachnoid, and subarachnoid space. Only very few patients with cancer have LMC. In the practice of general surgeons, this diagnosis is rarely, if ever, encountered. We present a rare case of a patient presenting to ED with worsening headaches over several months that developed acute-onset abdominal pain while being evaluated. Further workup showed free air, and the patient was taken emergently to the OR, where a perforated gastric ulcer was identified and biopsied. Pathology revealed gastric adenocarcinoma and subsequent MRI pointed to suspected LMC. Unfortunately, till today there is no effective treatment for advanced-stage gastric cancer, and aggressive intrathecal chemotherapy is only available to mitigate leptomeningeal involvement.

## Introduction

Leptomeningeal carcinomatosis (LMC) or leptomeningeal metastasis is defined as metastasis to the pia mater, arachnoid, and subarachnoid space. It is reported that 5% to 15% of patients with cancer have LMC [1]. The prognosis of LMC is poor due to limited therapeutic options, with an average survival of only two to six months [2,3]. LMC can happen to both central nervous system (CNS) tumors and non-CNS cancers. The malignant tumor cells can seed the leptomeninges through blood, cerebrospinal fluid (CSF), choroid plexus, or brain parenchyma. The current therapeutic strategy mainly includes whole-brain or cranial-spinal radiation and intrathecal or systemic chemotherapy [4-6]. However, a limited effect is achieved due to medication toxicity and the blockage by the blood-brain barrier. The main clinical presentations of LMC include cranial nerve dysfunction, inflammation, and hydrocephalus.

The current guideline indicates that patients with high Karnofsky scores, normal neurologic functions, and limited systemic disease have the best prognosis with multimodal interventions, including whole-brain radiation therapy and CNS penetrant systemic therapy [7]. Systemic chemotherapy covering the entire neuroaxis is also recommended for extra-CNS disease control because tumor cells may migrate with CSF flow and circulate from the choroid plexus to other locations, such as the sacral subarachnoid space.

## Case presentation

**Figure 1:**
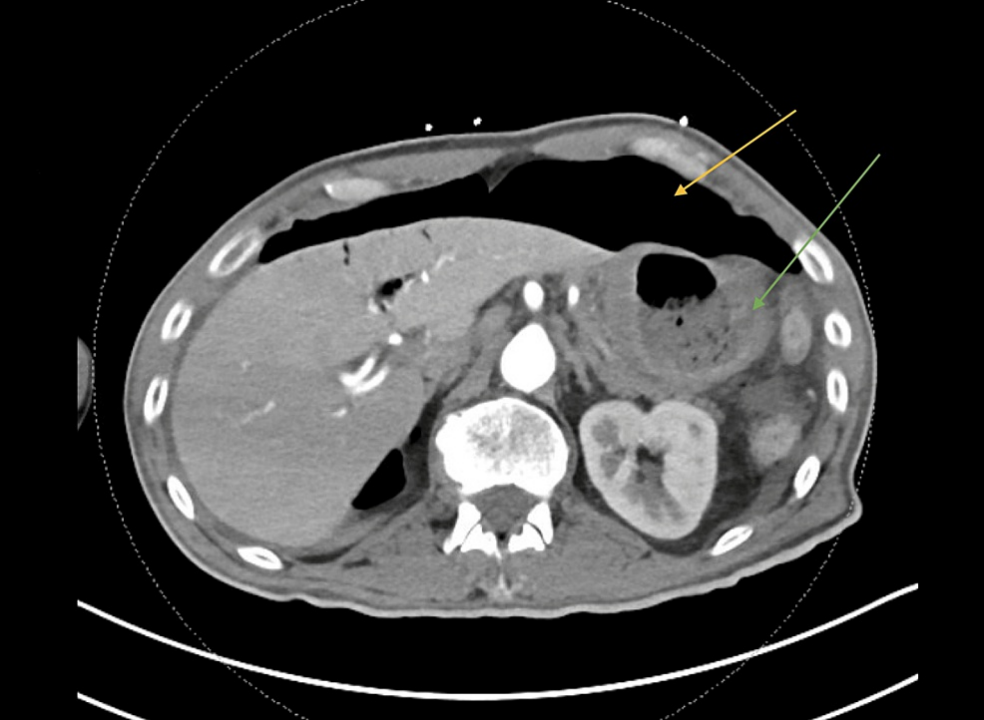
CT scan of the abdomen with intravenous contrast showing large-volume peritoneum Large-volume peritoneum is shown by yellow arrow and stomach wall thickening is shown by green arrow. CT, computed tomography.

**Figure 2:**
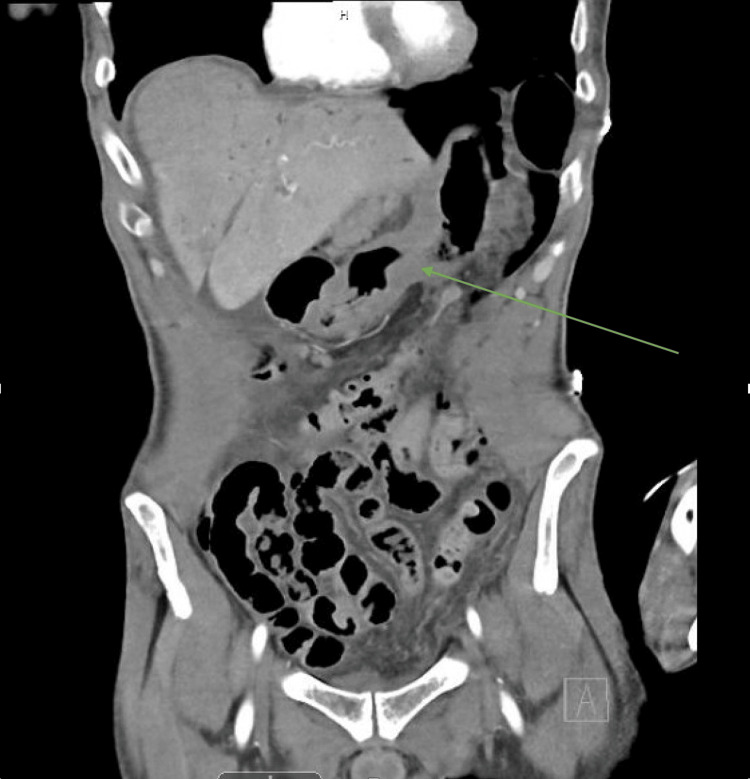
CT scan of the abdomen and pelvis with intravenous contrast Stomach wall thickening is shown by green arrow. CT, computed tomography.

We present a case of a 53-year-old male with a history of several months of worsening headaches and gastroesophageal reflux disease (GERD), who presented to the emergency room (ER) with a severe headache episode. He underwent computed tomography (CT) of the head that was negative for acute findings. However, shortly into his ER stay, the patient started having severe abdominal pain. On physical examination, the patient had diffuse tenderness to palpation and rebound tenderness consistent with peritonitis. Laboratory workup showed leukocytosis as well as lactic acidosis. Furthermore, an imaging workup, including a CT of the abdomen and pelvis (Figure 1), showed pneumoperitoneum and thickening of the stomach wall (Figures 1, 2). The patient was resuscitated with intravenous fluids, started on broad-spectrum antibiotics and antifungals, and taken to the OR for diagnostic laparoscopy.

**Figure 3:**
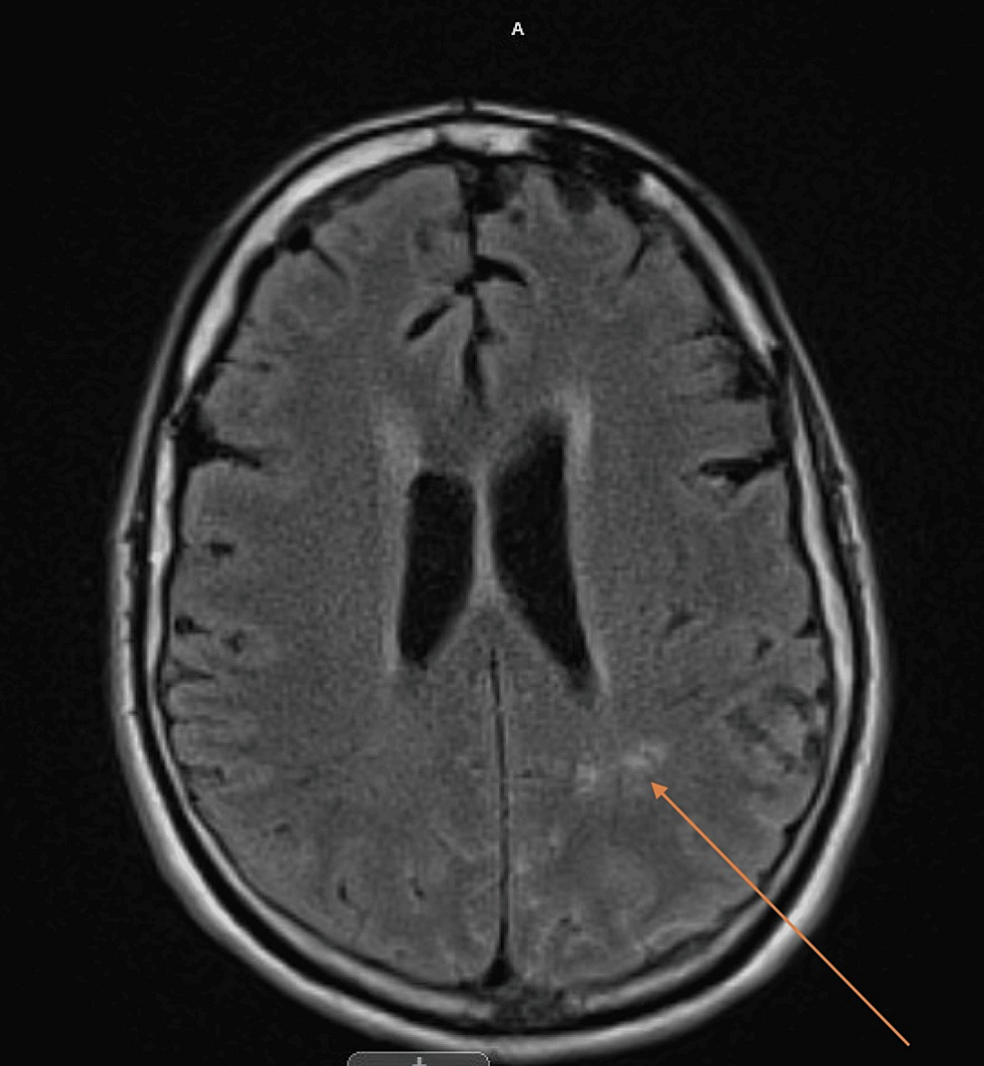
MRI FLAIR window showing subtle meningeal enhancement Meningeal enhancement of the occipital lobe is shown by orange arrow. MRI, magnetic resonance imaging; FLAIR, fluid-attenuated inversion recovery.

He was found to have gastric perforation with minimal intraperitoneal contamination. Intraoperative findings were concerning for underlying malignancy, but no diffuse metastatic disease was identified during the diagnostic laparoscopy. Postoperatively the patient was kept nil per os (NPO) with nasogastric tube decompression. On postoperative day (POD) 3, the upper gastrointestinal study showed no leak and the patient was started on a clear liquid diet. On POD 5, he was advanced to a soft diet. Follow-up pathology showed poorly differentiated gastric adenocarcinoma. Immunohistochemistry was positive for pan keratin and negative for HER2 mutation. An initial CT scan of the abdomen and pelvis was reviewed and did not show any metastatic disease. Due to persistent and worsening headaches, the patient initially underwent repeated CT head, which was negative, followed by an MRI and lumbar puncture with CSF analysis. The MRI showed subtle meningeal enhancement in the occipital lobe (Figure 3).

These findings were consistent with LMC. Malignant cells in the CSF confirmed the diagnosis of LMC. Due to the unfortunate poor prognosis of such results, the patient had a port placed and was started on aggressive intrathecal chemotherapy with methotrexate. Unfortunately, the patient did not tolerate the therapy well and after two months without any notable improvement, the family opted for hospice care. 

## Discussion

LMC is a rare late-stage complication in patients diagnosed with cancer caused by metastases to the leptomeninges, which are pia mater, arachnoid space, and subarachnoid space [8]. Patients with cancers such as breast cancer, non-small cell lung cancer, and gastrointestinal cancer are commonly known to present with LMC in later stages of diagnosis [8,9]. This is an unusual diagnosis in the case of a general surgeon. Patients who present with perforated gastric malignancy are usually advanced stage III or IV.

About 1%-8% of cancer patients in the United States are diagnosed with LMC [8]. LMC is a rare occurrence with gastric cancers but has been reported to be associated with 0.16%-0.19% of gastric cancers [10]. The most common subtype of gastric cancer associated with LMC is poorly differentiated adenocarcinoma with signet ring cell features [11]. 

Patients may present with visual disturbances, headache, diplopia, or cauda equina syndrome, depending on the location of metastasis [8,12]. Patients show signs of communicative obstruction, i.e., vomiting, positional headaches, and nausea due to malignant growth that would impair the flow of CSF [8].

Patients who come in with such clinical symptoms may be dismissed as they could be mistaken for signs and symptoms of metastatic disease, but immediate intervention is needed in these patients as symptoms progress quickly.

A diagnosis is made through CSF cytology positive for malignant cells, protein level >45 mg/dl, CSF pressure >150 mm, CSF pleocytosis, and glucose level <60 mg/dl. A T1-weighted magnetic resonance imaging with gadolinium contrast showed pial enhancement and nodularity over the cerebral convexities, in the basal cisterns or the ventricular ependymal surfaces and patchy involvement with occasional matting and intradural extramedullary nodules mostly at the cauda equina [8,9].

Management of perforated gastric ulcers in general is with Graham's patch - placing a tongue of omentum to provide vascularized coverage, whether with (modified Graham's patch) or without closure of the perforation. Management of a perforated gastric cancer with laparoscopy, as opposed to the open approach, should allow for a rapid recovery and return to baseline performance status as well as permit the onset of appropriate treatment.

Treatment of LMC is mainly done through intrathecal chemotherapy consisting of methotrexate, cytarabine, and steroids, whole-brain radiation [11], and palliative care [12].

## Conclusions

LMC presents a diagnostic challenge without localization of the primary tumor. Patients who present with perforated gastric cancer are automatically considered to have T4 disease and are frequently in stage III or IV, which carries a poor prognosis. A rare diagnosis of LMC has an overall dismal prognosis, and most patients will not survive past several weeks. Further studies are needed to determine whether the laparoscopic approach to repair perforated gastric cancer is beneficial as it allows faster recovery and rapid initiation of treatment, and might improve patient outcomes.
